# Gene-Expression Analysis of Human Fibroblasts Affected by 3D-Printed Carboxylated Nanocellulose Constructs

**DOI:** 10.3390/bioengineering10010121

**Published:** 2023-01-16

**Authors:** Jennifer Rosendahl, Chiara Zarna, Joakim Håkansson, Gary Chinga-Carrasco

**Affiliations:** 1 Unit of Biological Function, Division Materials and Production, RISE Research Institutes of Sweden, P.O. Box 857, SE-50115 Borås, Sweden; 2 RISE PFI, Høgskoleringen 6b, 7491 Trondheim, Norway; 3 Department of Laboratory Medicine, Institute of Biomedicine, Gothenburg University, P.O. Box 440, SE-40530 Gothenburg, Sweden

**Keywords:** wound dressings, nanocellulose, characterization, 3D-printing, gene expression

## Abstract

Three-dimensional (3D) printing has emerged as a highly valuable tool to manufacture porous constructs. This has major advantages in, for example, tissue engineering, in which 3D scaffolds provide a microenvironment with adequate porosity for cell growth and migration as a simulation of tissue regeneration. In this study, we assessed the suitability of three cellulose nanofibrils (CNF) that were obtained through 2,2,6,6-tetramethylpyperidine-1-oxyl (TEMPO)-mediated oxidation. The CNFs were obtained by applying three levels of carboxylation, i.e., 2.5, 3.8, and 6.0 mmol sodium hypochlorite (NaClO) per gram of cellulose. The CNFs exhibited different nanofibrillation levels, affecting the corresponding viscosity and 3D printability of the CNF gels (0.6 wt%). The scaffolds were manufactured by micro-extrusion and the nanomechanical properties were assessed with nanoindentation. Importantly, fibroblasts were grown on the scaffolds and the expression levels of the marker genes, which are relevant for wound healing and proliferation, were assessed in order to reveal the effect of the 3D-scaffold microenvironment of the cells.

## 1. Introduction

Cellulose nanofibrils (CNF) obtained through 2,2,6,6-tetramethylpyperidine-1-oxyl (TEMPO)-mediated oxidation are among the most frequently studied cellulosic materials for biomedical applications [1,2,3,4,5]. Specifically, ultrapure TEMPO CNFs were tested against wound pathogens (Gram-negative *Pseudomonas aeruginosa* and Gram-positive *Staphylococcus aureus*) and their antibacterial properties were demonstrated [6]. In addition, Nordli et al. [7,8] confirmed the biocompatibility of ultrapure TEMPO CNF, which they tested against human fibroblasts, keratinocytes, and in direct contact with blood cells in a human whole-blood model.

Three-dimensional (3D) printing is a technology for the production of well-defined objects, through the controlled deposition of ink. Hence, 3D-printing is considered a promising technology for tailor-made wound dressings with controlled structures and compositions, adapted to the specific needs of the patients [9]. TEMPO CNF hydrogels have several suitable properties for use as ink for 3D-printing by micro-extrusion (as well as denominated direct-ink-writing), such as their shear-thinning properties and high zero-shear viscosity [3,4,9,10,11,12,13,14]. Recent reviews on the 3D (bio)printing of nanocellulose-based inks have indicated the potential of specific CNF for biomedical applications [10,15,16], and TEMPO CNF appears as a promising material due to its good rheological properties at low concentrations.

In addition to the rheology, the mechanical performance of gels, and especially of 3D-printed constructs, might affect the responses of cells. The material strength and stiffness affect the cell behavior in cell cultures; for instance, certain phenotypes of cells might be induced by certain material stiffnesses [17]. The cell-growth rate and cellular spreading were shown to decrease with increasing material stiffness [18]. To control the stiffness, the crosslinking density of the substrate matrix and molecular interactions can be adjusted, additional constituents (e.g., CNF) can be incorporated, and architectural designs can be applied by using fabrication approaches, such as 3D-printing [19]. Hydrogels from natural materials were shown to have stiffnesses in the range of 0.3 kPa to over 1000 kPa [19]. The elastic stiffness of hydrogels is typically measured by atomic-force microscopy, nanoindentation [20], or universal testing machines [19]. Nanoindentation is widely used to determine the mechanical properties of soft biological tissue. Compared to compression tests using universal testing devices, only very small sample sizes are required, and the test conditions are easier to control [21]. Using a nanoscale indentation tip, the local surface stiffness and inhomogeneities can be assessed [21]. For cell migration, the local surface stiffness might be more relevant than the bulk stiffness because cell migration depends on traction forces that induce deformations in the order of micrometers [22]. The reduced elastic modulus (*E*_r_) obtained from nanoindentation generally includes the responses of the specimen and the test device itself [23]. In addition, the hardness is the ability of a material to resist local deformation and is about three times higher than the ultimate tensile strength of a material [24]. Nanoindentation is considered one of the most relevant testing methods to assess the mechanical properties of hydrogels [21]. This is because it is non-destructive, repetitive tests can be performed on only one sample, and the experimental accuracy is improved compared to compression tests [21].

Pre-clinical trials for functionality and safety, evaluated in relevant in vivo environments using animal models, are often necessary to bring a wound product to market. However, new regulations and legal requirements focus on 3R, i.e., reducing, refining, and replacing animal testing in the development of new treatments. Therefore, when relevant in vitro models, mimicking the in vivo situation, are available, they should preferably be used, and new sophisticated cell models based on 3D-printed constructs are promising systems for replacing some forms of animal testing [25].

An adequate tool used In combination with 3D cell models is gene-expression analysis. The mRNA expression in cells is quickly shifted and the turnover time is often within minutes [26], revealing the responses of cells upon exposure to a given chemical or microenvironment, such as a biomaterial scaffolds. Fibroblasts are active in the wound-healing process; allowing these cells to attach and grow on a scaffold material and analyzing the expression levels of marker genes relevant to different steps in the wound-healing process can contribute to an understanding of the cell response as it relates to the exposure of a material/microenvironment. The wound-healing process contains different phases and cell responses, including hemostasis, inflammation, proliferation, and remodeling [25] and, depending on the nature of the wound healing (acute or chronic), different genetic pathways are induced or reduced [27,28].

To create these 3D-models, hydrogels based on TEMPO CNF are potential candidates as they have high water-retention capacity and can create soft and porous structures in which cells can grow. Hence, the purpose of this study was to assess the printability of TEMPO CNFs that were obtained with different carboxyl acid contents, causing varying nanofibrillation. We hypothesized that TEMPO CNF with increasing nanofibrillation and at low concentrations would have an adequate viscosity and shear-thinning behavior for the 3D-printing of scaffolds for cell growth. This was demonstrated in this study, and the effect of the 3D-printed scaffolds on human fibroblasts was assessed through the corresponding quantification of the cells’ gene expressions.

## 2. Materials and Methods

### 2.1. Production and Characterization of CNF Grades

The CNFs with varying surface chemistries were produced through TEMPO-mediated oxidation [29], applying three oxidation levels, i.e., 2.5, 3.8, and 6.0 mmol hypochlorite (NaClO)/g cellulose, defined as CNF_2.5, CNF_3.8, and CNF_6.0, respectively. The CNFs were homogenized in a Rannie-15-type 12.56X homogenizer (1000 bar pressure) and the materials were collected after 3 passes. A detailed description of the CNF production was reported in our previous study [30]. Some key characteristics of the produced CNFs are reproduced in Table 1.

### 2.2. Viscosity of the CNFs

Viscosity was measured at various speeds and using spindle V-73 (temperature of 23 ± 1 °C), in a Brookfield viscometer (Brookfield DV2TRV, AMETEK Brookfield, Middleboro, MA, USA).

### 2.3. Three-Dimensional-Printing Trials and Characterization

The three CNF grades (concentrations 0.2 wt% and 0.6 wt%) were tested for 3D-printing, performed with a Regemat3D printing unit. For each series (CNF_2.5, CNF_3.8, and CNF_6.0), four constructs (dimensions 20 mm × 40 mm × 2 mm) were printed using a 0.58-mm printing nozzle and a flow speed of 3 mm/s. The spaces between the printed tracks were 2 mm × 2 mm and the height (2 mm) was composed of 4 printed layers.

As an additional test of print fidelity, the printing performances of the three CNF grades (CNF_2.5, CNF_3.8, and CNF_6.0) were assessed. Three replicates (20 mm × 40 mm) were printed, using a CNF concentration of 0.6 wt%. The structures were composed of only 1 layer for better assessment of printing performance, in which the distance between printed tracks was 2 mm and the flow speed 3 mm/s.

Images of the 3D-printed structures were acquired immediately after printing with an Epson Perfection V750 PRO scanner (Epson, Suwa-shi, Nagano, Japan), in transmission mode, and the applied resolution was 2400 dots per inch. The transmission of light through the optical images was quantified with the ImageJ program (version 1.52h) and is reported as the fraction of light transmitted through the construct, relative to the background.

The 3D-printed structures were frozen at −20 °C and freeze-dried as described above. Scanning electron microscopy (SEM) assessment of the freeze-dried samples was performed with a Hitachi SU3500 Scanning Electron Microscope (Hitachi, Tokyo, Japan). The gold coating was applied with an Agar Auto Sputter Coater (Agar Scientific, Essex CM24 8GF UK). Images were acquired in secondary electron imaging (SEI) mode using 5 kV and 6 mm of acceleration voltage and working distance, respectively. The texture orientation of the images was quantified with the SurfCharJ plugin for ImageJ [31].

### 2.4. Nano-Mechanical Assessment of 3D-Printed Constructs

Grids were printed in two layers 20 mm in diameter and 1 mm in height using a 0.58-mm nozzle and a flow speed of 3 mm/s. The grids were immersed in CaCl_2_ (100 mM) for at least 24 h before mechanical assessment with a TI950 Triboindenter (Bruker, former Hysitron, Billerica, MA, USA). The nanoindentation parameters were: conical tip; displacement controlled at the peak indentation depth of 2000 nm; 0.125 s loading, 0.4 s holding, 0.125 s unloading (total testing time 0.65 s for one indent). At least 20 reproducible indents on random areas were undertaken for each sample.

### 2.5. Gene-Expression Analysis

**Figure 1:**
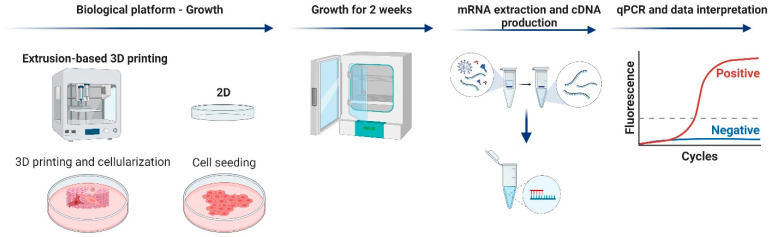
Gene-expression-analysis process. TEMPO CNF scaffolds were 3D-printed using an extrusion printer. Detroit551 fibroblasts were added to the scaffolds and cultured for 2 weeks. As a control, cells were cultured in conventional plastic cell culture dishes. The mRNA was extracted from the cells; reverse transcription was performed to produce cDNA and qPCR was performed with marker genes for wound healing and proliferation.

Human fibroblast cells Detroit551 (ATCC CCL-110™, Manassas, VA, USA) were cultured on 2D culture plates or on 3D-printed scaffolds, and the expression levels of marker genes known to be regulated by wound healing (selected from the SAB target list for wound healing) were compared) Figure 1.

#### 2.5.1. Cell Culture

The fibroblasts were kept sub-confluent in a minimum essential medium supplemented with fetal bovine serum 10%, 1X non-essential-amino-acid solution (NEAA), 1X sodium pyruvate, 1X GlutaMAX, and penicillin–streptomycin 1X (Gibco Life Technologies, Carlsbad, CA, USA), and cultured at 37 °C with 5% CO_2_. Cell suspensions were prepared by washing cells in Dulbecco’s phosphate buffered saline (DPBS) without calcium and magnesium (Gibco Life Technologies, Carlsbad, CA, USA) and by detaching cells using Trypsin (2.5%) without phenol red (Gibco Life Technologies, Carlsbad, CA, USA). Cell pellets were obtained by washing cells in medium (as above) and centrifuged at 300× *g* for 3 min. The pellets were then resuspended in medium. A 24-well plate was prepared with medium, in which the 3D-printed scaffolds were placed for 1 h. The medium was removed, and cells were seeded at a cell density of 150,000 cells/mL in a total volume of 2 mL and cultured at 37 °C at 5% CO_2_. The scaffolds were cultured for 24 h, after which they were moved to 6-well plates (TPP, Trasadingen, Switzerland). The medium was changed every 3–4 days by moving the scaffolds to new 6-well plates (TPP, Trasadingen, Switzerland) for a total culturing time of 2 weeks. Cells cultured in 2D were seeded in 6-well plates (TPP, Trasadingen, Switzerland) at a cell density of 250,000 cells per well and cultured for 72 ± 3 h. Light-microscopy images were taken of the cells (Supplementary Figure S1) before harvesting of the cells and RNA extraction.

#### 2.5.2. RNA Extraction and qPCR

Gene expression was examined using quantitative PCR (qPCR) on extracted RNA from fibroblasts, as follows. Scaffolds were rinsed in DPBS (Gibco Life Technologies, Carlsbad, CA, USA) and moved to a 2-milliliter low-binding tube prepared with 700 µL QIAzol (Qiagen, Hilden, Germany). The tubes were snap-frozen in liquid nitrogen and stored at −80 °C until further analysis. After the samples were thawed at 2–8 °C, they were disrupted using 5-mm stainless-steel beads (Qiagen, Hilden, Germany) and TissueLyser II (Qiagen, Hilden, Germany) for 2 × 2.5 min at 25 Hz. The RNA was extracted using a miRNA micro kit (Qiagen, Hilden, Germany) with on-column DNase digestion (Qiagen, Hilden, Germany) according to the manufacturer’s instructions, except that prior to adding the sample to the extraction column, the supernatant was not mixed with ethanol. Nanodrop ND-1000 (Thermo Fisher Scientific, Waltham, MA, USA) was used to determine the total nucleic-acid concentration. Complementary DNA (cDNA) was generated using an iScript cDNA Synthesis Kit (Bio-Rad, Hercules, CA, USA) in 20-microliter reactions and a CFX96 thermal cycler (Bio-Rad, Hercules, CA, USA) at 22 °C for 5 min, 42 °C for 30 min, and 85 °C for 5 min followed by cooling to 4 °C. The cDNAs were diluted 1:10 in RNase-free water (Invitrogen). The qPCRs were run in 10-microliter reactions using 400-nanometer concentrations of primer (Bio-Rad, Hercules, CA, USA) (Supplementary Table S1) and 1 X SSO Advanced Universal SYBR Green Supermix (Bio-Rad, Hercules, CA, USA) in a CFX384 Touch Real Time Cycler (Bio-Rad, Hercules, CA, USA) at 95 °C for 30 sec, with 45 cycles of amplification at 95 °C for 10 s, 60 °C for 30 s with an acquisition point, followed by a melting-curve analysis at 65 °C to 95 °C with 0.5 °C per 5-s increment. Cycles of quantification (Cq) values were determined by the regression method using CFX Manager software version 3.1 (Bio-Rad, Hercules, CA, USA) and analyzed using GenEx v6 (MultiD, Gothenburg, Sweden). Missing values were imputed based on replicates; an average of qPCR replicates was used, a cut-off was set at Cq-value 35, and the remaining missing values were assigned Cq-value 35. All values were normalized to reference genes identified with the NormFinder algorithm (YWHAZ), transformed to relative values to two dimensions (2D) and values were then log2. To analyze the pattern of the expression of all genes together and find out any potential clustering, principal component analyses (PCA-plots) were generated using the GenEx Software (MultiD, Gothenburg, Sweden). One graph generated the summary of all gene expressions, identifying each sample in the PCA plot (gene-expression plot), and one graph was generated to identify genes that affected each sample in the PC1/PC2 directions (gene load).

### 2.6. Statistical Analysis

The statistical analysis software SPSS (IBM) and GraphPad Prism (Prism, San Diego, CA, USA) were used to calculate the significance in a one-way ANOVA with two different post hoc tests, Tukey HSD and Bonferroni.

## 3. Results and Discussion

### 3.1. Three-Dimensional Printing

**Figure 2:**
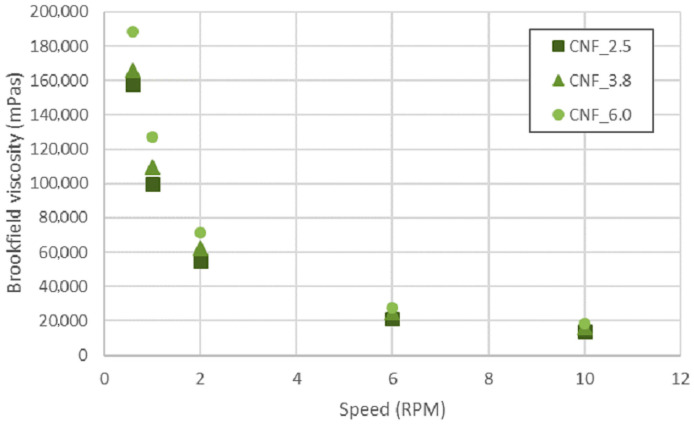
Brookfield viscosity assessment, measured at various speeds.

The rheological characteristics reported for some CNFs are beneficial for 3D-printing [32,33,34]. In this study, the CNF_6.0 had a higher viscosity than samples CNF_2.5 and CNF_3.8 (Table 1). This was due to the apparently large fraction of individualized nanofibrils in sample CNF_6.0 [30], which caused a corresponding increase in viscosity (Table 1 and Figure 2). The three samples showed a reduction in viscosity as the speed increased, which can be explained by the shear-thinning effect.

Shear thinning facilitates the deposition of CNF inks on a substrate during printing and the rapid recovery of zero-shear viscosity implies that 3D-printed structures do not collapse after deposition. High viscosity at relatively low concentrations is due to the large entanglement of individual nanofibrils [35]. Notably, the pKa of TEMPO CNF has been reported to be 3.6 [36]; thus, the CNF gels were ionized at pH 6.5 in this study.

**Figure 3:**
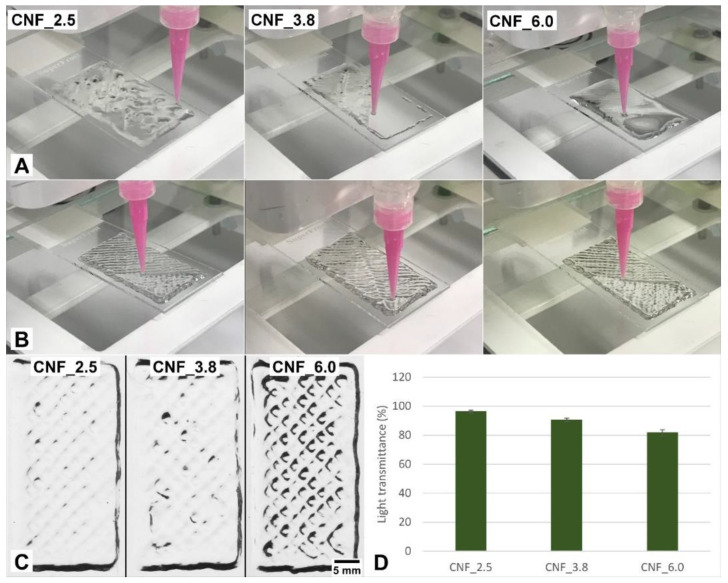
Three-dimensionally printable inks. (**A**) Exemplification of poor printability due to low CNF concentration (0.2 wt%). (**B**) Improved printability of 3D constructs with CNF at a concentration of 0.6 wt%. Left, middle, and right correspond to CNFs 2.5, 3.8, and 6.0 mmol, respectively. (**C**) Optical images of the 3D-printed constructs in four layers, CNF concentration = 0.6 wt%. (**D**) Quantification of light transmittance of 3D-printed constructs. Staples represent average and error bars; standard deviation, n = 4. The target dimensions of the 3D-printed constructs were 20 mm × 40 mm × 2 mm.

The effect of the CNF concentration on the print resolution is illustrated in Figure 3. Note that the printed structures collapsed when the concentration of the CNF ink was low, i.e., 0.2 wt% (Figure 3A). This was expected, since a low concentration of CNF (<0.2 wt%) leads to low viscosity, which has been reported to be almost constant over a wide range of shear rates [37]. Regarding TEMPO CNF, a concentration above 0.5 wt% leads to a pronounced shear-thinning behavior and increased viscosity at a low shear rate [37]. This was confirmed in the present study, in which the TEMPO CNFs (concentration 0.6%) showed shear-thinning behavior and high viscosity at low shear. These characteristics facilitated an adequate 3D-printing process, i.e., the deposited tracks apparently did not collapse, and the 3D constructs were printed (Figure 3B,C).

Images of the 3D constructs (target dimensions 40 mm × 20 mm × 2 mm) were acquired and the light transmittance was quantified (Figure 3C,D). Note the weaker definition of the printed tracks in samples CNF_2.5 and CNF_3.8 compared to CNF_6.0, showing a better print fidelity. The quantification of the light transmittance through the constructs confirms the observed differences. The higher light transmittance of CNF_2.5 and CNF_3.8 was presumably due to the lateral flow of the CNF dispersion after printing, i.e., the printed pattern was not maintained. It should be kept in mind that the viscosity of the samples was CNF2.5 < CNF_3.8 < CNF_6.0 (Table 1). Low viscosity leads to a lateral flow of the CNF after it is deposited on a substrate, i.e., the structure may have a tendency to collapse as layers are printed on top of each other. The CNF_6.0 sample demonstrated a 3D construct with well-defined tracks, which is an indication of good print fidelity (Figure 3C).

**Figure 4:**
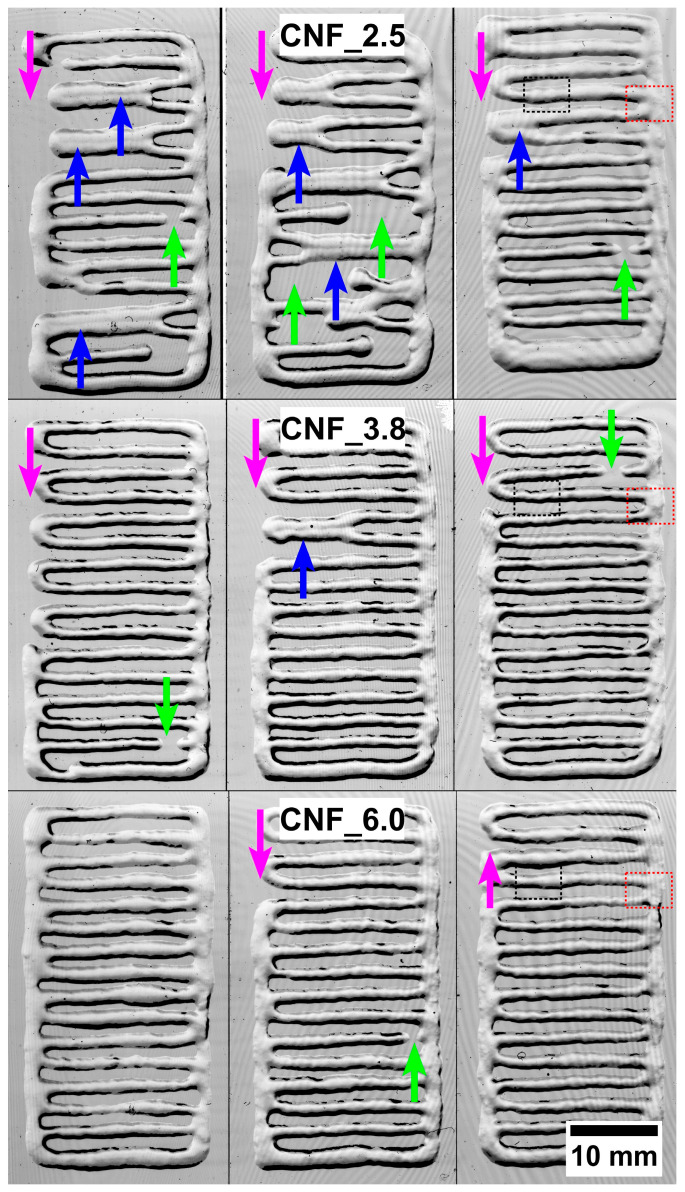
Evaluation of printability of the inks CNF_2.5, CNF_3.8, and CNF_6.0. The three columns provide three corresponding replicates for each series. The major defects were observed in structures printed with CNF_2.5, including missing edges (magenta arrows), the fusing of printed tracks (blue arrows), and empty spaces (green arrows). The target size of the printed structures was 20 × 40 mm in one layer. The black and red dotted rectangles on the right side indicate the areas for horizontal and vertical SEM analyses, respectively (Figure 5). The contrast of the images was improved for better visualization.

To further analyze and compare the printability of the three assessed CNFs, constructs composed of only one layer were printed (Figure 4). The differences in printability were confirmed as the number of defects decreased with the higher CNF surface charge (carboxyl acid content, Table 1). The defects were as follows: (i) printed tracks that tended to collapse because of the flow and merging neighboring tracks (blue arrows), (ii) zones with empty spaces in the inner areas (green arrows), and (iii) areas with empty spaces at the edges (magenta arrows). The lateral flow and the merging of neighboring tracks can be attributed to the relatively low viscosity of samples CNF_2.5 and CNF 3.8 (Figure 4). However, the empty spaces were most probably caused by a larger occurrence of micrometer-sized residual fibers in sample CNF_2.5. Residual fibers may cause agglomeration and clog the nozzle, thus limiting the extrusion of the ink. The CNF_6.0 sample had a relatively high viscosity and, thus, a larger fraction of nanofibrils (Figure 3 and Table 1), contributing to improved printability, resulting in the absence of major defects in the printed structures (Figure 4).

### 3.2. Structural and Mechanical Analysis

**Figure 5:**
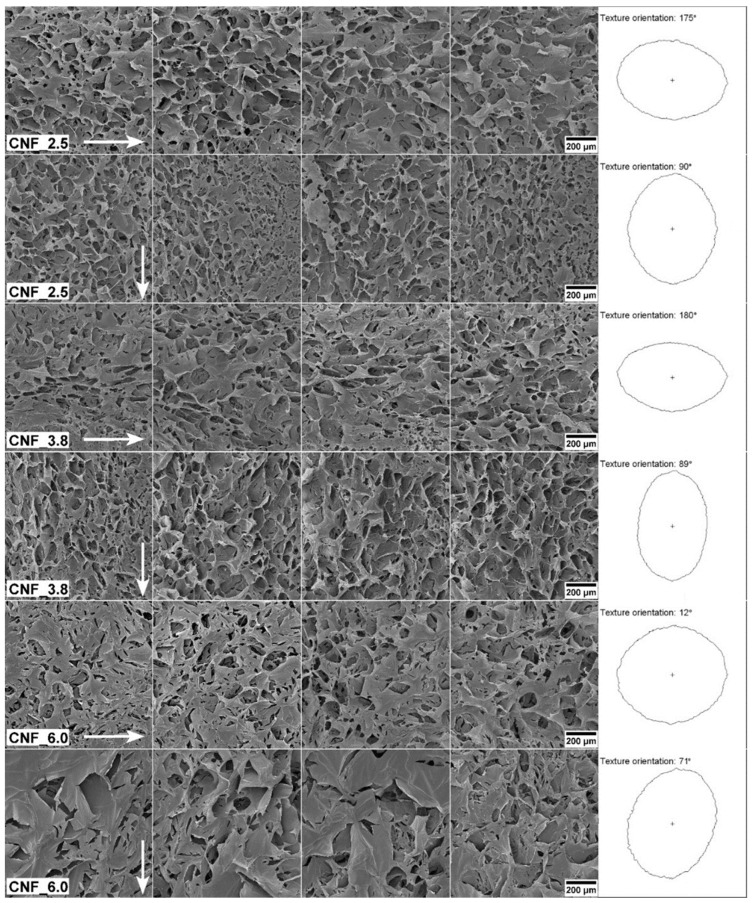
SEM assessment of freeze-dried constructs. The four columns provide four replicate SEM images for each series. The arrows indicate the printing direction. The right-column polar plots illustrate the main orientation of the surface structure.

The 3D-printed structures were freeze-dried and assessed with SEM to explore their pore structures (Figure 5). The analysis indicated pore sizes at the micrometer scale, ranging from roughly 10 μm to 200 μm. Particular characteristics of CNF are the high aspect ratio of the individualized nanofibrils and the length in the micrometer scale compared to the nanometric cross-sectional dimensions [38]. Facilitated by these characteristics and the shear forces during extrusion, the nanofibrils aligned in the printing direction, as was demonstrated previously for cellulose nanocrystals [39]. The alignment of individual nanofibrils seems to affect the self-assembly of the structure after lyophilization (Figure 5). Using computerized gradient analysis based on Sobel operators [40,41], we were able to quantify the orientation of the aerogel’s texture. This is represented by polar plots of azimuthal facets, which indicate the main direction of the orientation [31]. The more elongated the polar plot, the more pronounced the orientation in a given direction. The polar plots of the structures printed in a horizontal direction are horizontally oriented, compared to the vertically oriented polar plots of the structures printed vertically. The CNF_3.8 had clear orientation patterns defined by the micrometer-sized surface pores, while the CNF_6.0 exhibited a more isotropic texture. The surface texture of CNF_6.0 was composed of flakes/walls of self-assembled nanofibrils and the flakes observed on the images of sample CNF_6.0 resembled the film layers formed on rough surfaces (Figure 5). previously reported by Ottesen et al. [42] The authors attributed the formation of these films to the charge and specific surface area of TEMPO CNF, which increase the viscosity and affect the drying mechanism.

Controlling the orientation of the printed pattern is particularly interesting for scaffolds and tissue engineering to control the growth and proliferation of cells in a given direction. A clear example was reported by Wu et al. [43], who compared an aligned poly (L-lactic acid) scaffold with a corresponding random scaffold. The authors demonstrated that human-adipose-derived mesenchymal stem cells aligned, elongated, and proliferated more in the aligned structure compared with cells grown in the randomly fabricated scaffold. This is particularly interesting for mimicking the extracellular matrix structure of native tendons, thus facilitating the tissue engineering of tendon grafts [43].

**Figure 6:**
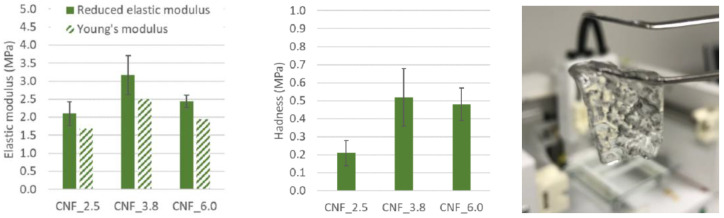
Nano-mechanical properties of CNF hydrogels (0.6 wt.%). (**Left**) Reduced and Young’s modulus. (**Middle**) Hardness. (**Right**) Example of a 3D-printed specimen cross-linked with CaCl_2_.

In addition to the orientation of the scaffolds, the stiffness is an important property to assess. Stiffness, the resistance to deformation (in the elastic region) of a material under an applied force, is important for the mechano-transduction response of cells [44]. For example, cells respond to the stiffness of biomaterials by reorganizing their cytoskeletons, affecting their spread, proliferation, and migration [45]. Thus, the stiffness of the biomaterial affects the biological behavior of the cells and tissue, which may be important from a wound-healing point of view. The surface indentation applied in this study ensured the hardness of the gels, which is not limited to the elastic region of a stress- and strain-curve. The stiffness and hardness of the CNF gels assessed in this study (concentration = 0.6 wt%) are reported in Figure 6. The results reveal the level of the elastic modulus, i.e., ~2–3 MPa, and the hardness (~0.2–0.5 MPa) of the three sets of gels. The hardness of gels for topical applications was described previously by Jones et al. [46], reporting an increase in hardness with increased concentrations of carboxymethylcellulose. Polyelectrolyte complexes were previously reported to have a higher level of hardness, compared to neat polysaccharides applied to the treatment of wounds [47]. Note the 3D-printed CNF gel that was cross-linked with Ca^2+^ and that is capable of withstanding its own weight (Figure 6, right).

The values of the elastic modulus are given in Figure 6 (left) agree with those in a study on TEMPO CNF hydrogels with concentrations of 1% (*w*/*v*) in water [48]. Films with 0.5% CNF were previously shown to yield a tensile modulus of approximately 2 MPa and a tensile strength of approximately 0.17 MPa [49], which is similar to the Young’s modulus, and approximately a third of the hardness [24] obtained by nanoindentation in this study. The Young’s modulus in Figure 6 (left) was calculated using Equation (S1) (Supplementary information). Using macroscale compression tests, the elastic modulus of comparable hydrogels was shown to be more than 10 times lower than the presented values [17,50]. This could be related to the difference in the measurement of local material properties with nanoindentation and global material and structural properties in compression tests. The difference in scale makes it possible to include or exclude structural properties, e.g., porosity or 3D-printed grid patterns. However, the comparison of elastic moduli measured with different testing techniques is challenging due to the nonlinear behavior at larger strains of hydrogels [51], strain-rate dependence, and possible tension–compression anisotropy [52]. Nanoindentation measurements are influenced by both the compression and the tension moduli of the material [52].

Since sample CNF_2.5 showed poor 3D printability (Figure 4), only CNF_3.8 and CNF_6.0 were used for the cell culture and gene-expression evaluation. We previously confirmed that these materials were neither cytotoxic nor causes of skin irritation [30].

### 3.3. Cell Culture and Gene-Expression Analysis

The microenvironment composition has a very large impact on the cell characteristics of a tissue. We previously showed that the heterogeneity of tumor-tissue microenvironments affects the mRNA and protein expression of breast- and colon-cancer cells repopulating decellularized tumor tissue [53,54]. By adapting material characteristics and using 3D-printing, we have a promising opportunity to develop synthetic scaffolds for cell cultures to mimic human-tissue microenvironments. We recently showed that alginate- and nanocellulose-based hydrogels can be used for the 3D-printing of scaffolds simulating breast-cancer tumors and that breast-cancer cells grown in scaffolds adopt gene-expression profiles that are more similar to in vivo situations than cells grown in conventional 2D cultures [48,55]. In the present study, the Detroit551 fibroblast cell line was used to study the microenvironment’s effect on the wound-healing properties. Fibroblasts are cells present in soft tissues with the function of maintaining the structural integrity of connective tissue by producing and remodeling extracellular matrix (ECM) molecules [56]. Since ECM components are important components of wound healing that influence cell survival, proliferation, and function, fibroblasts play a critical role in the wound-healing process [57]. Detroit551 fibroblasts are embryonically derived and are therefore different from adult fibroblasts in terms of late-wound-healing capabilities, such as scar-tissue formation and proliferation status [58]. Although the interpretation of our data may be limited by these characteristics, this cell line is frequently used in the wound-healing studies in the literature [59,60,61,62,63,64,65], and here, we report the early signs of the effects on wound healing as adaptations of cells to the microenvironment of 3D-printed TEMPO CNF scaffolds. While fibroblasts cultured in 2D plastic culture dishes grow in a monolayer, they grew in three dimensions in multiple layers in our 3D-printed scaffolds (Supplementary Figure S1). In a study performed by Pereira et al. [66], cotton-cellulose nanofibers were shown to affect the viability of bovine fibroblasts negatively at a concentration above 2000 µg/mL (viability < 70%, which is the limit of cytotoxicity set by the ISO 10993-5 standard for medical devices). This effect was probably due to the physical impact of the nanofibers on the cells, and cells in an in vitro monolayer are much more sensitive to physical stress compared with cells in tissue. The increase in the gene-expression levels of the stress-response genes, *HSP70.1*, *PRDX1*, and *BAX* at a nanofiber concentration above 2000 µg/mL is also a logical response to the decreased viability.

**Figure 7:**
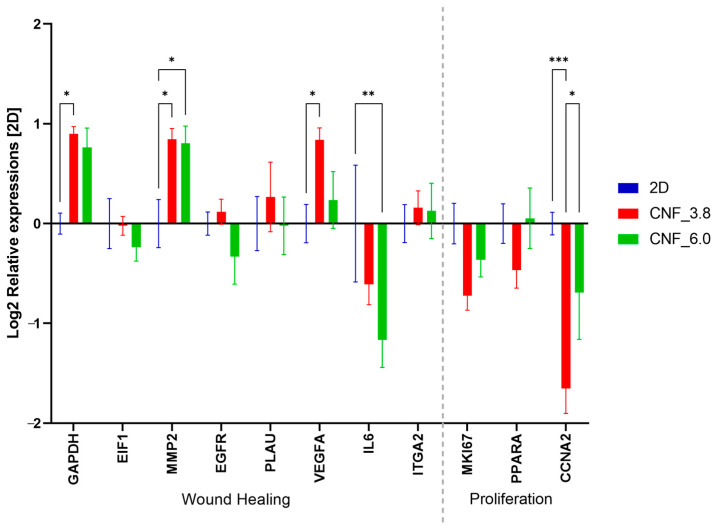
The microenvironment of 3D-printed TEMPO CNF scaffolds affects the expression of genes related to wound healing and proliferation. Gene expression changed on fibroblasts cultured for 2 weeks in 3D-printed TEMPO CNF scaffolds and 72 h in 2D were analyzed by qPCR. Relative expressions were adjusted to the gene expression of the 2D control, set to zero for each gene. Staples represent the average and error bars standard error of the mean (n = 9). Two-way ANOVA; Tukey’s post hoc test for multiple comparisons (2D). * *p*-value < 0.05, ** *p*-value < 0.01, *** *p*-value < 0.001.

In this study, the gene-expression analysis was used to analyze how the microenvironment of the 3D-printed TEMPO CNF scaffolds affected the cell characteristics of the fibroblasts. The cells were cultured in 3D-printed TEMP CNF scaffolds for 2 weeks and their gene-expression profiles were compared with those of the cells cultured in 2D, and a confluence of approximately 80% was reached (72-h culture). Next, the cells in 2D grew confluent and shifted from a growth-active state to a cell-arrest state with low activity, while the cells in the 3D scaffolds continued to grow in multiple layers. In previous studies on cell cultures in 3D-printed scaffolds, we found that after 2–3 weeks (depending on the cell type), the cells adapted to the 3D microenvironment and the gene-expression profile can be adequately analyzed. 3D-printed scaffolds affect cell growth, for example, through the reduced proliferation of cancer cells in favor of other important in vivo cell characteristics, such as cancer stemness and migration, compared with conventional 2D cell growth [55]. Here, marker genes relevant to wound healing and proliferation were chosen for the expression analysis. All the proliferation markers (*MKI67*, *PPARA*, and *CCNA2* (statistically significant)) showed a trend of downregulation in the cells grown in 3D-TEMPO CNF scaffolds compared with the cells grown in 2D, except for *PPARA* in CNF_6.0, where it was unaffected (Figure 7). The proliferation markers were all expressed in the nucleus, with different functions. The marker of proliferation (*MKI67*) covering the chromosomes is associated with mitosis [67] and is widely used in histology. Peroxisome proliferator activated receptor alpha (*PPARA*) is a transcription factor regulating the peroxisomal beta-oxidation of fatty acids. Cyclin A2 (*CCNA2*) functions as a regulator of the cell cycle in encouraging cell-cycle transitions in the G1/S and G2/M phases.

Of the wound-healing-marker genes, *EIF1*, *EGFR*, *PLAU*, and *ITGA2* were not statistically significantly changed compared with the 2D cultures. Eukaryotic initiation factors (*EIF’s*) are fundamental for the translation of mRNA to protein by ribosomes and thereby affect the function of many other genes [68]. The epidermal growth factor receptor (*EGFR*) is involved in epidermal and dermal regeneration and is a stimulator of proliferation [69]. The plasminogen activator, urokinase (*PLAU*), is involved in blood coagulation and fibrinolysis [70], and the Integrin subunit alpha 2 (*ITGA2*) is involved in the adhesion of platelets and other cells to collagens, the modulation of collagen and collagenase gene expression. Glyceraldehyde-3-phosphate dehydrogenase (*GAPDH*), which has a key function in glycolysis, is commonly used as a house-keeping gene, as well as in wound-healing studies [71]; however, in our study, we found that *GAPDH* was upregulated in the cells grown in the 3D-printed scaffolds compared with 2D. The matrix metallopeptidase 2 (*MMP2*) and vascular endothelial growth factor A (*VEGFA*) were both significantly upregulated compared with the cells in the 2D culture. The gene *MMP2* is expressed by fibroblasts for the reorganization of extracellular matrix and *VEGFA* is functional as a stimulator of angiogenesis, vasculogenesis, cell migration, granulation and scar tissue formation, and endothelial cell growth. Interleukin 6 (*IL6*) was the only wound-healing marker that was significantly downregulated compared with the 2D culture. The gene *IL6* is a cytokine with a wide variety of biological functions in immunity and tissue regeneration, and it is a key modulator of the inflammatory and reparative process. The expression of *IL6* is activated upon wound induction, and the downregulation of this gene in our model might be explained by the absence of tissue or cell damage. In a general trend, the cells grown in the CNF_3.8 scaffolds had lower expression levels of proliferation markers and higher expression levels of wound-healing markers compared with the cells grown in the CNF_6.0 scaffolds (Figure 7).

**Figure 8:**
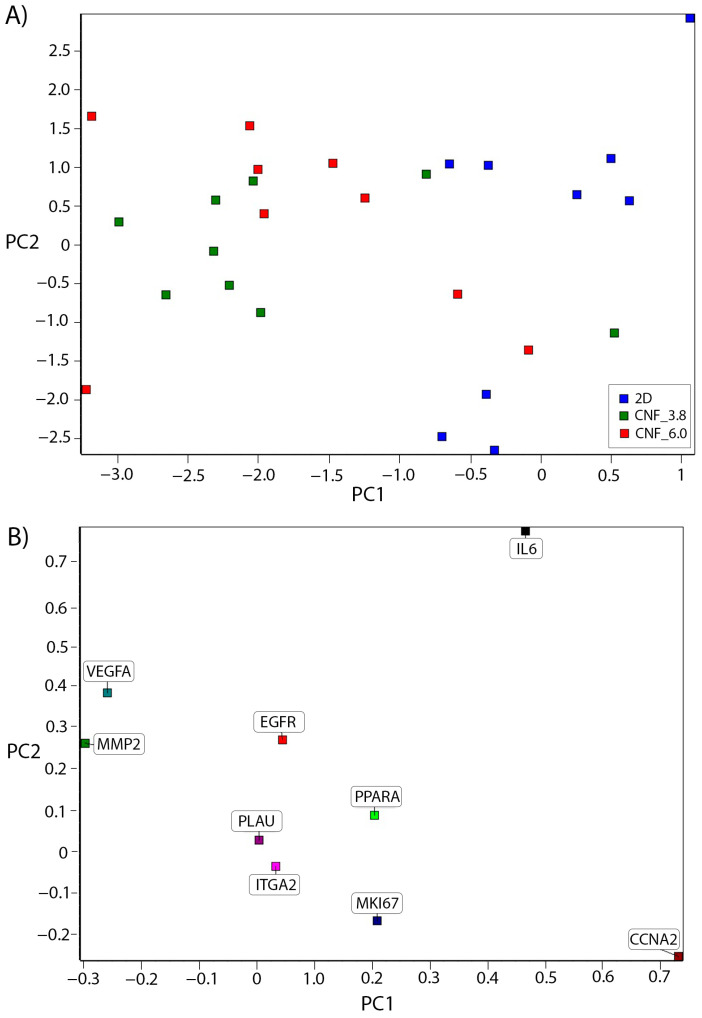
Principal component analysis of gene-expression data is presented as sample scores (**A**), where each square represents a replicate sample, and gene loading, where each square represents a gene (**B**). Blue is 2D, Green is CNF_3.8, and Red is CNF_6.0.

Principal component analysis (PCA) is a useful tool with which to identify patterns of complex data sets. In this case, it was used for the expression-level analysis of several genes. By performing a statistical multivariate analysis, the PCA plot clustered the samples in relation to the expression levels of the marker genes used. Our analysis showed that the qPCR results of the cells grown in TEMPO CNF_3.8 (clustered to the left in Figure 8A) deviated the most from the cells cultured in 2D (clustered to the right in Figure 8A), while the samples from cells grown in the TEMPO CNF_6.0 scaffolds were scattered in between the other groups (Figure 8A). A gene-loading plot (Figure 8B) illustrates how much each gene influenced the PCA. The genes influencing the PCA plot, affecting the samples to the right, were the proliferation marker CCNA2 and IL-6, whilst VEGFA and MMP2 affected the samples towards the left side of the PCA plot. These results suggest that the microenvironment of the TEMPO CNF_3.8 3D-printed scaffolds affected the fibroblasts more than the 2D-cultured cells.

## 4. Conclusions

The printability of the three CNF grades was evaluated and we concluded that the more nano fibrillated the material, the higher the viscosity and the better the 3D-printing performance, as demonstrated by the micro-extrusion and computerized image analysis of the 3D-printed constructs. The deposition of the CNF gels by micro-extrusion yielded an anisotropic orientation of the porous structure, presumably caused by the preferred orientation of the nanofibrils during the extrusion. The fibroblasts grown in the 3D-printed TEMPO CNF scaffolds showed a downregulation of proliferation marker genes, which was in line with our previous observations of cancer cells grown in 3D-printed scaffolds, in which proliferation was downregulated in 3D growth compared with 2D. The most significantly affected wound-healing-marker genes were correlated with the stimulation of ECM reorganization (*MMP2*) and vascularization (*VEGFA*), whereas the expression of the inflammatory marker, *IL6*, was suppressed. The multivariate analysis of the gene expression concluded that the TEMPO CNF_3.8 scaffold microenvironment had a larger influence on the fibroblast growth in terms of the expression of proliferation and wound-healing-marker genes than the 2D-cultured cells. The 2D cell culture had more similarities with CNF_6.0 than with CNF_3.8. This suggests that the oxidation level of CNF_3.8 (with a carboxylic acid content of ~1.3 mmol/g) is more adequate for wound-healing dressings or models used for patient and screening platforms.

## Figures and Tables

**Table 1:** CNF characterization. Values reproduced from [30].

Analyses	CNF_2.5	CNF_3.8	CNF_6.0
pH (0.6 wt%)	6.81 ± 0.02	6.83 ± 0.02	6.89 ± 0.01
Carboxylic-acid content (μmol/g)	1036 ± 41	1285 ± 42	1593 ± 10
Viscosity at 10 RPM (mPas)	13,855 ± 17	18,157 ± 25	18,208 ± 35
Mean object size (nm)	708	509	498

## Data Availability

Data is available upon request.

## Supplementary Materials

The following supporting information can be downloaded at: https://www.mdpi.com/article/10.3390/bioengineering10010121/s1, Equation S1 calculation of by Young’s modulus, Figure S1: Light microscopy images of Detroit551 fibroblasts; Table S1: Primer sequences. Ref. [72] has been cited in the Supplementary Materials.
