# Supplementary material for: Gene-Expression Analysis of Human Fibroblasts Affected by 3D-Printed Carboxylated Nanocellulose Constructs
Source: Bioengineering (Basel). 2023 Jan 16;10(1):121. doi: 10.3390/bioengineering10010121 (PMC9854960; doi:10.3390/bioengineering10010121)
Supplement: Supplementary file 1 [file bioengineering-10-00121-s001.zip › bioengineering-2132803-supplementary.pdf]

Young's modulus of the specimen material E_s can be calculated taking the material properties of the indenter material (the Young's modulus E_i and the Poisson's ratio ν_i of *e.g.,* diamond) into account [1]:

$$E_s = \frac{1 - {\nu_s}^2}{\frac{1}{E_r} - \frac{1 - {\nu_i}^2}{E_i}} \quad \text{(S1)}$$

The Young's modulus in **Figure 6** (left) was calculated using Equation (S1). Material properties of the diamond indentation tip were taken from literature [2] and a Poisson's ratio of 0.45 was assumed for the hydrogel material.

1. Li, X.; Bhushan, B. A review of nanoindentation continuous stiffness measurement technique and its applications. *Mater. Charact.* **2002**, *48*, 11–36. https://doi.org/10.1016/S1044-5803(02)00192-4.

2. Klein, C.A.; Cardinale, G.F. Young's modulus and Poisson's ratio of CVD diamond. *Diam. Relat. Mater.* **1993**, *2*, 918–923, https://doi.org/10.1016/0925-9635(93)90250-6.

Supplementary Table S1. Primer sequences

**Assay Information**	PrimePCR™ SYBR® Green Assay: MMP2, Human
**Technology:**	qPCR
**Assay Type:**	SYBR® Green
**Application:**	Gene Expression
**Unique Assay ID:**	qHsaCID0015623
**Assay Design:**	Intron-spanning
**Chromosome Location:**	16:55519623-55522561
**Amplicon Length:**	144
**Splice Variants Targeted:**	ENST00000219070, ENST00000437642, ENST00000543485

**Assay Information**	PrimePCR™ SYBR® Green Assay: GAPDH, Human
**Technology:**	qPCR
**Assay Type:**	SYBR® Green
**Application:**	Gene Expression
**Unique Assay ID:**	qHsaCED0038674
**Assay Design:**	exonic
**Chromosome Location:**	12:6647267-6647413
**Amplicon Length:**	117
**Splice Variants Targeted:**	ENST00000229239, ENST00000396856, ENST00000396861, ENST00000396859, ENST00000396858

**Assay Information**	PrimePCR™ SYBR® Green Assay: PPARA, Human
**Technology:**	qPCR
**Assay Type:**	SYBR® Green
**Application:**	Gene Expression
**Unique Assay ID:**	qHsaCID0011001
**Assay Design:**	Intron-spanning
**Chromosome Location:**	22:46594443-46611188
**Amplicon Length:**	135
**Splice Variants Targeted:**	ENST00000420804, ENST00000407236, ENST00000402126, ENST00000396000, ENST00000262735, ENST00000434345, ENST00000535613

**Assay Information**	PrimePCR™ SYBR® Green Assay: EIF1, Human
**Technology:**	qPCR
**Assay Type:**	SYBR® Green
**Application:**	Gene Expression
**Unique Assay ID:**	qHsaCID0010867
**Assay Design:**	Intron-spanning
**Chromosome Location:**	17:39846397-39847113
**Amplicon Length:**	95
**Splice Variants Targeted:**	ENST00000469257

**Assay Information**	PrimePCR™ SYBR® Green Assay: CCNA2, Human
**Technology:**	qPCR
**Assay Type:**	SYBR® Green
**Application:**	Gene Expression
**Unique Assay ID:**	qHsaCID0017452
**Assay Design:**	Intron-spanning
**Chromosome Location:**	4:122742135-122743624
**Amplicon Length:**	149
**Splice Variants Targeted:**	ENST00000274026

**Assay Information**	PrimePCR™ SYBR® Green Assay: MKI67, Human
**Technology:**	qPCR

**Assay Type:**	SYBR® Green
**Application:**	Gene Expression
**Unique Assay ID:**	qHsaCID0011882
**Assay Design:**	Intron-spanning
**Chromosome Location:**	10:129910624-129911742
**Amplicon Length:**	108
**Splice Variants Targeted:**	ENST00000368654, ENST00000368653, ENST00000537609, ENST00000368652

**Assay Information**	PrimePCR™ SYBR® Green Assay: VEGFA, Human
**Technology:**	qPCR
**Assay Type:**	SYBR® Green
**Application:**	Gene Expression
**Unique Assay ID:**	qHsaCED0006937
**Assay Design:**	Exonic
**Chromosome Location:**	6:43745206-43745349
**Amplicon Length:**	114
**Splice Variants Targeted:**	ENST00000372067, ENST00000324450, ENST00000417285, ENST00000413642, ENST00000372055, ENST00000482630, ENST00000425836, ENST00000372064, ENST00000372077, ENST00000519767, ENST00000520948, ENST00000523873, ENST00000523950, ENST00000457104, ENST00000518689, ENST00000523125, ENST00000518824, ENST00000230480, ENST00000520265

**Assay Information**	PrimePCR™ SYBR® Green Assay: PLAU, Human
**Technology:**	qPCR
**Assay Type:**	SYBR® Green
**Application:**	Gene Expression
**Unique Assay ID:**	qHsaCID0019740
**Assay Design:**	Intron-spanning
**Chromosome Location:**	10:75675057-75676271
**Amplicon Length:**	196
**Splice Variants Targeted:**	ENST00000372764 ,ENST00000372761, ENST00000372762, ENST00000446342

**Assay Information**	PrimePCR™ SYBR® Green Assay: EGFR, Human
**Technology:**	qPCR
**Assay Type:**	SYBR® Green
**Application:**	Gene Expression
**Unique Assay ID:**	qHsaCID0007564
**Assay Design:**	Intron-spanning
**Chromosome Location:**	7:55221808-55223611
**Amplicon Length:**	97
**Splice Variants Targeted:**	ENST00000455089, ENST00000342916, ENST00000344576, ENST00000420316, ENST00000275493, ENST00000442591, ENST00000454757, ENST00000395504, ENST00000533450

**Assay Information**	PrimePCR™ SYBR® Green Assay: IL6, Human
**Technology:**	qPCR
**Assay Type:**	SYBR® Green
**Application:**	Gene Expression
**Unique Assay ID:**	qHsaCID0020314
**Assay Design:**	Intron-spanning
**Chromosome Location:**	7:22768389-22769247
**Amplicon Length:**	122
**Splice Variants Targeted:**	ENST00000404625, ENST00000426291, ENST00000401651, ENST00000407492, ENST00000401630, ENST00000406575, ENST00000258743, ENST00000420258

**Assay Information**	PrimePCR™ SYBR® Green Assay: YWHAZ, Human
**Technology:**	qPCR
**Assay Type:**	SYBR® Green
**Application:**	Gene Expression
**Unique Assay ID:**	qHsaCID0013897
**Assay Design:**	Intron-spanning
**Chromosome Location:**	8:101932945-101936435
**Amplicon Length:**	175
**Splice Variants Targeted:**	ENST00000395957, ENST00000395956, ENST00000353245, ENST00000522542, ENST00000521309, ENST00000517797, ENST00000522819, ENST00000395953, ENST00000395948, ENST00000395951, ENST00000419477, ENST00000521607, ENST00000457309, ENST00000395958

**Assay Information**	PrimePCR™ SYBR® Green Assay: ITGA2, Human
**Technology:**	qPCR
**Assay Type:**	SYBR® Green
**Application:**	Gene Expression
**Unique Assay ID:**	qHsaCID0016134
**Assay Design:**	Intron-spanning
**Chromosome Location:**	5:52358616-52360776
**Amplicon Length:**	149
**Splice Variants Targeted:**	ENST00000296585

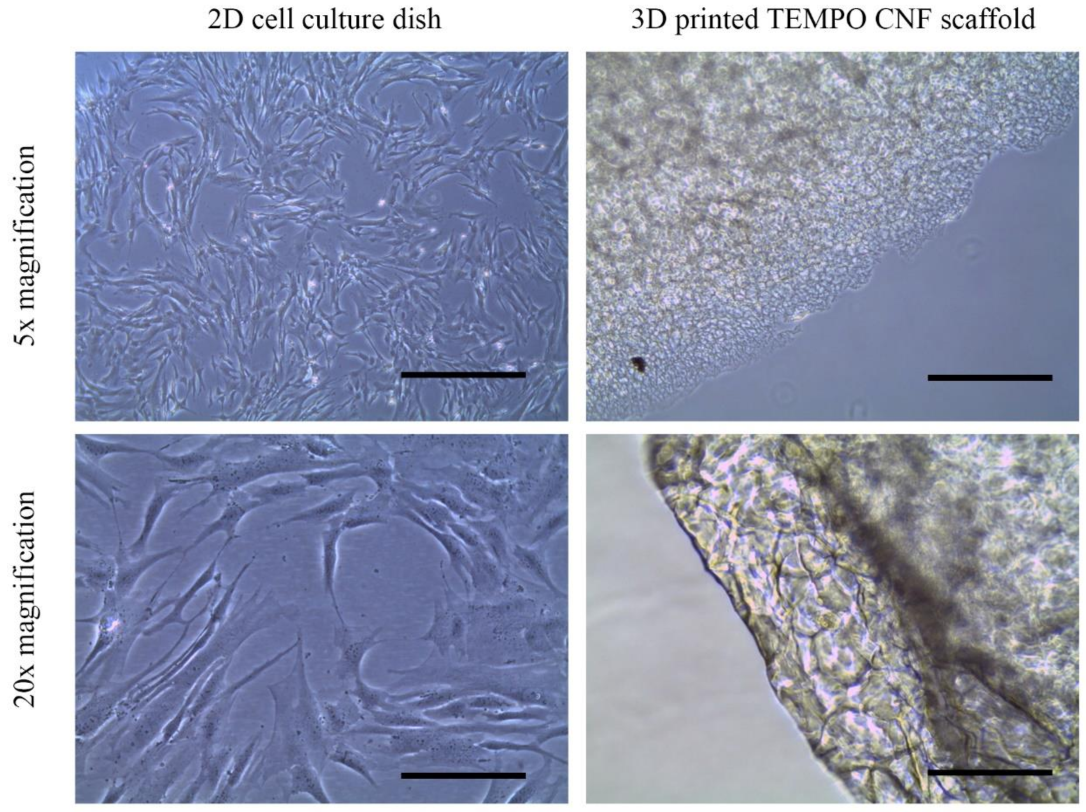

Supplementary Figure S1: Detroit551 fibroblasts grown for 72h days in 2D cell culture dishes (left column) or 2 weeks in 3D printed TEMPO CNF scaffolds (right column). Light microscopy images were taken at 5x (upper row) and 20x (lower row) magnification. Since the cells in the scaffold grow in multiple layers and in 3 dimensions, the visualization of the cells is not optimal. Scale bars in 20x magnifications are 20 μm and in 5x magnification 100 μm.
